# RasGRPs Are Targets of the Anti-Cancer Agent Ingenol-3-Angelate

**DOI:** 10.1371/journal.pone.0072331

**Published:** 2013-08-21

**Authors:** Xiaohua Song, Ana Lopez-Campistrous, Lucy Sun, Nancy A. Dower, Noemi Kedei, Jing Yang, Jessica S. Kelsey, Nancy E. Lewin, Tim E. Esch, Peter M. Blumberg, James C. Stone

**Affiliations:** 1 Department of Biochemistry, University of Alberta, Edmonton, Alberta, Canada; 2 Department of Pediatrics, University of Alberta, Edmonton, Alberta, Canada; 3 Laboratory of Cancer Biology and Genetics, Center for Cancer Research, National Cancer Institute, National Institutes of Health, Bethesda, Maryland, United States of America; H.Lee Moffitt Cancer Center & Research Institute, United States of America

## Abstract

Ingenol-3–angelate (I3A) is a non-tumor promoting phorbol ester-like compound identified in the sap of *Euphoria peplus.* Similar to tumor promoting phorbol esters, I3A is a diacylglycerol (DAG) analogue that binds with high affinity to the C1 domains of PKCs, recruits PKCs to cellular membranes and promotes enzyme activation. Numerous anti-cancer activities have been attributed to I3A and ascribed to I3A’s effects on PKCs. We show here that I3A also binds to and activates members of the RasGRP family of Ras activators leading to robust elevation of Ras-GTP and engagement of the Raf-Mek-Erk kinase cascade. In response to I3A, recombinant proteins consisting of GFP fused separately to full-length RasGRP1 and RasGRP3 were rapidly recruited to cell membranes, consistent with direct binding of the compound to RasGRP’s C1 domain. In the case of RasGRP3, IA3 treatment led to positive regulatory phosphorylation on T133 and activation of the candidate regulatory kinase PKCδ. I3A treatment of select B non-Hodgkin’s lymphoma cell lines resulted in quantitative and qualitative changes in Bcl-2 family member proteins and induction of apoptosis, as previously demonstrated with the DAG analogue bryostatin 1 and its synthetic analogue pico. Our results offer further insights into the anticancer properties of I3A, support the idea that RasGRPs represent potential cancer therapeutic targets along with PKC, and expand the known range of ligands for RasGRP regulation.

## Introduction

Diacylglycerol (DAG) is a potent second messenger that is generated in cells in response to membrane receptor stimulation of phospholipid metabolism. DAG and DAG analogues such as PMA (phorbol 12-myristate 13-acetate) bind conventional and novel forms of protein kinase C (PKC) through a conserved domain called C1. This process contributes to PKC membrane localization and enzyme activation. Prolonged exposure to DAG analogues can also negatively impact PKC activity through induced enzyme degradation. Some DAG analogues, such as PMA, are potent tumor promoters. Other DAG analogues, such as prostratin and bryostatin 1, are non-tumor promoters or may indeed inhibit tumor promotion. Medicinal DAG analogues such as bryostatin 1 exert a variety of anti-cancer cell and immune modulatory effects. Based on encouraging preclinical data, bryostatin 1 has been the subject of extensive cancer clinical trials (http://clinicaltrials.gov/ct2/results?term=bryostatin).

Another medicinal DAG analogue of clinical interest is ingenol-3-angelate (I3A). I3A was identified as an active agent in the sap of *Euphorbia peplus*, which has a history of use in traditional medicine, including the treatment of skin cancers [1]. Pep005, a topically applied formulation of I3A, is being vigorously evaluated in clinical trials (http://clinicaltrials.gov/ct2/results?term=pep005&pg=2). Pep005 has been shown effective in the treatment of actinic keratosis [2] and is being developed for the treatment of basal cell carcinoma and squamous cell carcinoma [1]. In different experimental models, I3A can induce tumor cell primary necrosis [3], apoptosis [4] and senescence [5]. Tumor cell-extrinsic anti-cancer effects of I3A include disruption of tumor vasculature [6], recruitment of tumoricidal neutrophils [7] and the generation of cytotoxic T cells with tumor regressing activity [8]. I3A binds to purified classical and novel PKC isoforms *in vitro* [9]. I3A treatment of cultured cells results in rapid relocation of these enzymes to cellular membranes [9]. Thus, some of the biological effects probably arise from I3A activation of PKCs in living cells.

Although early research on DAG and DAG analogues focused on their regulation of PKC family members, it is now clear that additional families of proteins exist with homologous DAG-binding C1 domains and that these families serve critical biological roles [10], [11]. The RasGRPs are positive regulators of the small GTPase Ras. The chimerins function as negative regulators of the small GTPase Rac, which regulates the actin cytoskeleton. MRCK (myotonic dystrophy kinase-related Cdc42 binding kinase) signals downstream of the small GTPase Cdc42, controlling filopodia formation and influencing tumor invasion. Munc13 family members regulate synaptic vesicle membrane fusion and synaptic activity. Protein kinase D family members play a key role in cellular proliferation and viability. DAG kinases convert DAG to phosphatidic acid, attenuating signaling through the above DAG-responsive protein families as well as potentially enhancing phosphatidic acid-responsive signaling pathways. Understanding the selectivity of therapeutic agents targeted to the DAG-binding C1 domains of these other classes of DAG-binding proteins is thus important for understanding their therapeutic potential and their mechanisms of action.

High-level expression of RasGRP1 and RasGRP3 shows restricted tissue distribution, with prominent expression in lymphocytes [12]. A growing body of evidence supports the hypothesis that a key role of these proteins is to function downstream of immune receptors in B and T cells [12]. Immune receptor stimulation leads to phospholipase C activation and DAG accumulation in membranes. DAG influences RasGRP1 and RasGRP3 through two coordinated mechanisms. First, the C1 domains of RasGRP1 and RasGRP3 bind DAG and phorbol ester [13], [14], causing the RasGRPs to be recruited to cellular membranes where they encounter their substrate, lipidated Ras. Deletion of the C1 domain causes loss of phorbol ester responsiveness [15]. Additionally, RasGRP1 and RasGRP3 undergo an activating phosphorylation by PKC, itself activated by DAG [16]–[19]. Ras activation by RasGRPs is important for thymocyte differentiation, T cell effector function, and lymphocyte homeostasis [12]. RasGRP4, like RasGRP1 and RasGRP3, also likely binds DAG and activates Ras [20], [21], although regulation by PKC has not been documented. RasGRP4 shows prominent expression in mast cells [21] but is also found in other myeloid lineages and early thymocytes [22], [23].

We have argued that RasGRPs might constitute viable drug targets that could be manipulated with DAG analogues to clinically beneficial ends [24]. In particular, we have shown that select cell lines derived from certain forms of B-non-Hodgkin’s lymphoma (B-NHL) undergo apoptosis when RasGRPs are stimulated with DAG analogues such as bryostatin 1 or the synthetic analogue “pico”. Initially, we focused on DAG analogue-induced apoptosis in the cell line Toledo, which likely arose from a germinal center-derived malignancy. We showed that apoptosis occurred within 48 hours and involved pro-apoptotic phosphorylation of the BH3-only protein Bim through the RasGRP-Ras-Raf-Mek-Erk pathway [25]. More recently, we discovered a second mechanism of induced apoptosis that was seen in a Burkitt’s lymphoma (BL)-derived cell line, BL-2, and in 5 of 6 mantle cell lymphoma (MCL)-derived cell lines [26]. Most of these cell lines were Bim-deficient and the apoptotic mechanism was associated instead with quantitative changes in other Bcl-2 family members: expression of the pro-apoptotic BH3-only family member Bik generally increased while that of the anti-apoptotic members Bcl-XL and Mcl-1 decreased. This second mechanism proceeded much more slowly than that observed in Toledo cells.

Here, we show that RasGRPs are targets of I3A in cultured cells. Furthermore, I3A treatment of representative B-NHL cell lines activates the two previously documented RasGRP coupled mechanisms leading to apoptosis.

## Materials and Methods

### Ethics Statement

Animal experiments were done according to protocols approved by the Health Sciences Animal Policy and Welfare Committee at the University of Alberta, in accordance with the Canadian Council on Animal Care Guidelines.

### Reagents

Ingenol-3-angelate was purchased from Calbiochem (La Jolla, CA) and was dissolved in DMSO at 1 mM. The stock was diluted to a final concentration of 100 nM in medium unless otherwise noted and compared to DMSO similarly diluted. Mek inhibitors and reagents for studying apoptosis were described previously [25], [26]. PMA was from LC laboratories (Woburn, MA), Gö6983, protease cocktail set 1 and NP-40 were from Calbiochem (Billerica, MA).

### Cell Culture

Rat2 cells were engineered to express the retrovirus vector pBabePuro or the vector derivatives containing cDNAs encoding RasGRP1, RasGRP3 or RasGRP4 as previously described [15]. Rat2 cells were cultured in DMEM containing 10% heat-inactivated fetal bovine serum supplemented with penicillin, streptomycin and glutamine (Gibco, Burlington, ON, Canada). Human lymphoma cell lines were cultured in RPMI-1640 (Gibco) supplemented as above. The origins of the cell lines have been published [25], [26]. The Ramos B cell line, LNCaP prostate cell line and HEK-293 cells expressing GFP-RasGRP3 [28] were grown in RPMI-1640 supplemented with 10% FBS and 2 mM glutamine (ATCC, Manassas, VA).

### Mice

*Rasgrp1−/−* mice have been previously described [27] and were maintained on the C57Bl/6J background. C57Bl/6J mice were used as wild type controls.

### I3A in vitro Binding Studies

Binding affinities of I3A to the RasGRP1 C1 domain and to RasGRP3 were determined as described previously [13], [14]. The incubation temperature, optimized for stability of the proteins under binding conditions, was 37°C for the RasGRP1 C1 domain and 18°C for RasGRP3.

### Analysis of Proteins by Immunoblotting

Analysis of active and total Ras, pErk1/2, Erk1/2 and Bcl-2 family members, unless otherwise noted, was described earlier [25], [26]. To analyze RasGRP3 phosphorylation, Ramos cells were treated with PMA, I3A or DMSO control vehicle as indicated for 30 minutes after which they were harvested in lysis buffer (1% NP-40 in PBS with protease inhibitor cocktail set 1). In some cases, cells were pretreated for 30 min with the pan-PKC inhibitor Gö6983 (5 µM). Immunoblotting was performed as described earlier [28] using the following antibodies: pRasGRP3T133 (Epitomics ab124823), RasGRP3 (Cell Signaling, #3334), PKCδ (Santa Cruz, sc-937), pPKCδ Ser299 (Epitomics ab133456), pErk1/2 (T202/Y204, Cell Signaling, #9106), Erk1/2 (Cell Signaling, # 9102), β-Actin (Santa Cruz, sc-47778). After development of the signals by ECL (enhanced chemiluminescence), the films were scanned and quantitation of the signal was performed using ImageJ (National Institutes of Health).

### Confocal Microscopy

Translocation of GFP-RasGRP1 in LNCaP cells was evaluated as described [29]. 60,000 LNCaP cells were plated on ibidi µ-dishes (Ibidi LLC, Verona, WI) and then transfected 48 h later with GFP-tagged RasGRP1-encoding plasmid using Lipofectamine reagent in combination with Plus reagent according to the manufacturer’s (Invitrogen, Carlsbad, CA) recommendations. After 24 hours, the cells were treated with 1000 nM of PMA or I3A in confocal medium (Dulbecco’s Modified Eagle Medium without phenol red supplemented with 1% FBS), and time-lapse images were collected every 30 s using the Zeiss AIM software. Imaging was with a Zeiss LSM 510 or Zeiss LSM 710 confocal microscopy system (Carl Zeiss, Inc.) with an Axiovert 100 M inverted microscope operating with a 25 mW argon laser tuned to 488 nm. A 63×1.4 NA Zeiss Plan-Apochromat oil-immersion objective was used together with varying zooms (1.4 to 2X). To create a vector for these studies hRasGRP1 (NM-005739) was inserted into the pQB125-fN1 vector by classical cloning using *Nhe*I restriction enzyme cleavage. The DNA sequences of the constructs were confirmed by sequence analysis (DNA, Minicore, CCR, NCI, NIH).

### Membrane Fractionation

HEK-293 cells expressing GFP-RasGRP3 [28] were treated with PMA or I3A (1000 nM or 100 nM) for 30 min or DMSO as vehicle control. At the end of the treatment the cells were washed in PBS, removed from plates by scraping and pelleted by low speed centrifugation (5000 RPM for 5 min in a refrigerated Eppendorf microfuge). Cells were sonicated (3×6 sec) in PBS supplemented with protease inhibitor cocktail set 1 and then centrifuged at low speed as above to remove unbroken cells. Aliquots of this supernatant representing “total protein” were reserved for analysis. The remainder of the supernatant was then subjected to high-speed centrifugation (100,000×g for 1 hr) to yield a supernatant “cytoplasmic fraction”. The high-speed pellet was washed, dissolved in 1% Triton-X 100, and detergent-insoluble material was removed by high-speed centrifugation, as above. The resulting final supernatant represents the “membrane fraction”. Fractions of total, cytoplasmic and membrane proteins were subjected to SDS polyacrylamide electrophoresis and immunoblotting, with equal protein loaded in each lane.

### Apoptosis and Cell Proliferation Assays

Methods for studying drug-induced apoptosis and the MTT assay for viable cell accumulation have been published [26].

## Results

### Binding of I3A to RasGRP1 and RasGRP3 in vitro

Both the RasGRP1 and the RasGRP3 C1 domains were previously shown to bind DAG analogues including phorbol esters [13], [14]. Modeling indicates that ingenol 3-esters bind to C1 domains in a similar fashion to phorbol esters and DAG [30]. Accordingly, we evaluated the binding of I3A using a competition assay using [^3^H]phorbol 12,13-dibutyrate, in the presence of 100 µg/ml phosphatidylserine (Table 1). RasGRP1 (C1 domain) and RasGRP3 (full-length) bound I3A with 2- and 9- fold greater affinity, respectively, than they bound the phorbol ester PDBu. Compared to PKCα, RasGRP1 and RasGRP3 bound I3A with approximately similar affinities, indicating that there was no selectivity between these two families of targets, at least *in vitro*.

**Table 1:** Comparison of binding affinities for ingenol 3-angelate (I3A) and phorbol 12,13-dibutyrate (PDBu).

	PKCα	RasGRP1 C1 domain	RasGRP3
***Ligand***	(K_d_, nM)	(K_d_, nM)	(K_d_, nM)
**Ingenol 3-angelate**	0.10±0.02*	0.073±0.005†	0.045±0.005†
**PDBu**	0.3±0.05†	0.12±0.01‡	0.42±0.03‡

* Value from [9].

† Values represent the mean ± SEM of four independent experiments.

‡ Value represents the mean ± SEM of three independent experiments.

### I3A Activates RasGRPs in Cultured Fibroblasts and Jurkat T Cells

**Figure 1:**
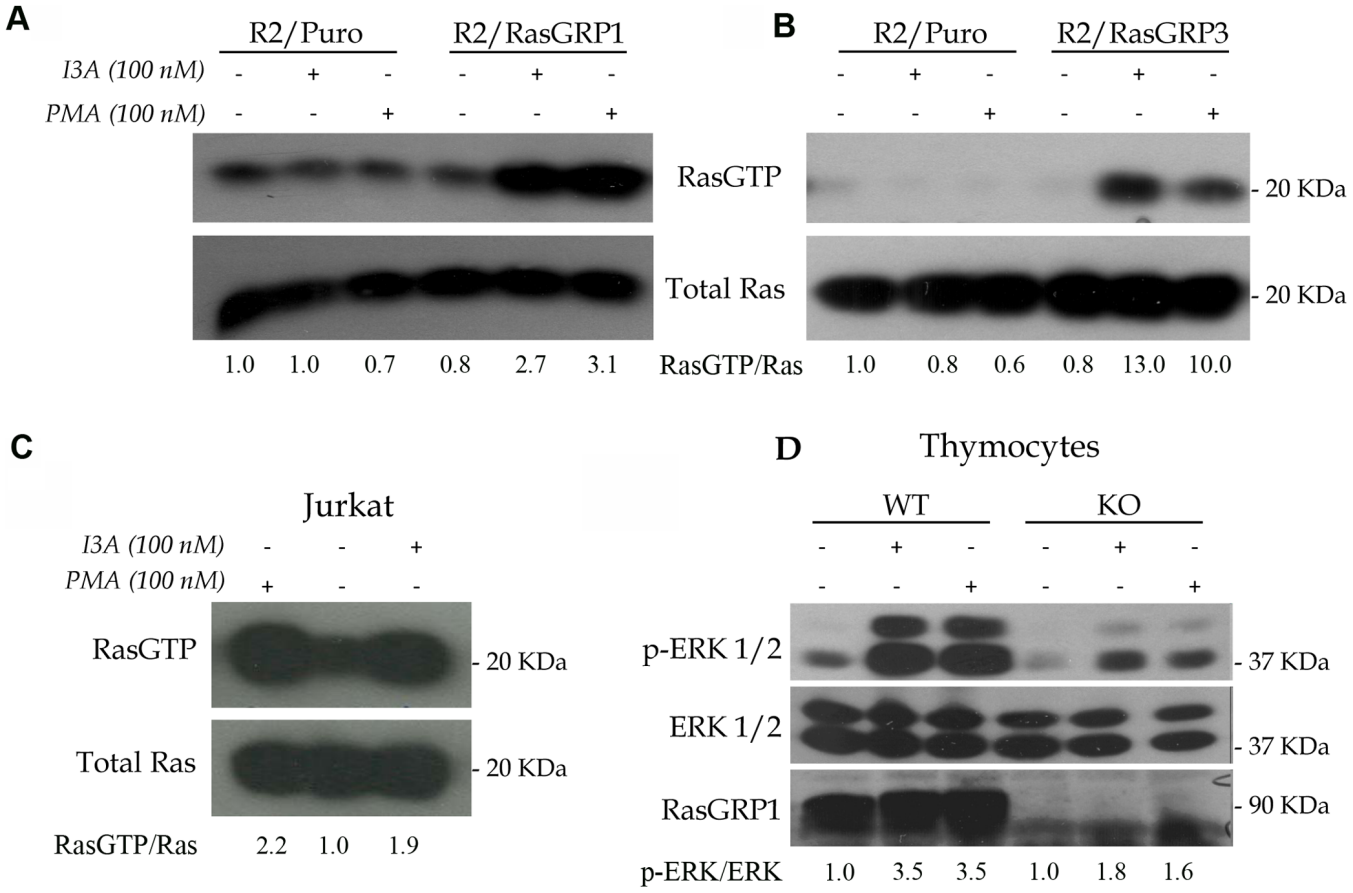
Activation of RasGRP1 and RasGRP3 by I3A. A. Cultures of Rat2 cells engineered with either empty vector or RasGRP1 cDNA-expressing vector were treated with I3A or PMA for 10 minutes and compared to control cultures using the Ras-GTP pull-down assay. Results are representative of three independent experiments. B. In a single experiment Rat2 cells expressing RasGRP3 cDNA were similarly analyzed. C. Jurkat T cells were analyzed for DAG analogue-induced Ras activation in three independent experiments. D. An equal number of C57Bl/6J (WT, wildtype) and *Rasgrp1*−/− (KO, knockout) thymocytes were treated with DMSO (negative control), I3A or PMA as indicated for 10 minutes and protein lysates were compared using the phospho-Erk signaling assay. The results are representative of duplicate experiments. Quantitation of the ratios of RasGTP/Ras or p-Erk1/2/Erk1/2 are presented below the individual panels.

Rat2 fibroblasts do not detectably express endogenous RasGRPs. Thus, Rat2 cells engineered to express individual RasGRPs, compared to cells engineered to express the empty vector, provide a convenient system to determine whether a DAG analogue can activate RasGRPs. Compared to cells expressing the empty vector, Rat2 cells expressing RasGRP1 showed robust Ras activation as assessed using a pull-down assay that recovered Ras-GTP bound to the Ras-binding domain of Raf1 (Fig. 1A). I3A was as effective as PMA in this assay. Likewise, expression of RasGRP3 in Rat2 cells conferred an ability to activate Ras in response to both DAG analogues (Fig. 1B). Jurkat T cells endogenously express RasGRP1 but not appreciable amounts of RasGRP3 or RasGRP4. I3A treatment reproducibly elicited reproducible Ras activation in Jurkat T cells, similar to that seen with PMA (Fig. 1C).

We previously showed that RasGRP1 was essential for DAG analogue-induced activation of Ras in mouse thymocytes; *Rasgrp1*−/− thymocytes were completely defective while wildtype (C57Bl/6J) thymocytes exhibited strong Ras activation [27]. Because we had limited numbers of cells, we used the more sensitive phospho-Erk assay as a surrogate for Ras activation. As expected, mutant thymocytes demonstrated much diminished signaling to Erk after either PMA or I3A treatment (Fig. 1D). Collectively, these results show that I3A, like PMA, can activate RasGRP1 and RasGRP3. A high basal Ras-GTP level in untreated cells frustrated similar experiments aimed at evaluating RasGRP4 in Rat2 cells. However, using a cell morphology assay we collected indirect evidence that I3A and PMA each can activate RasGRP4 in Rat2 cells. RasGRP4-expressing Rat2 cells incubated for 48 hours in PMA or I3A assumed a transformed morphology, as previously described for RasGRP1 expressing Rat2 cells [15]. This effect was not seen with empty vector cells or untreated RasGRP4-expressing Rat2 cells (data not shown).

DAG analogues activate RasGRPs by PKC-mediated phosphorylation as well as by direct recruitment to cellular membranes [16]–[19]. Using the Ramos B cell line, we determined the dose dependency of I3A and PMA for inducing phosphorylation of endogenous RasGRP3 on T133. In parallel, we examined both the activation of PKCδ, which could be assessed from its state of phosphorylation at S299 [31], as well as the downstream response of Erk phosphorylation. For kinases like PKC which are subject to allosteric and positional regulation, measurements of activity of the isolated kinase are not a reliable measure of the level of *in vivo* activity. For PKCδ, phosphorylation at S299 appears to report its ligand dependent activation [31]. Unfortunately, antibodies are not commercially available for phosphorylation sites reflecting the activation of other PKC isoforms, as distinct from the many antibodies reporting on phosphorylation of PKCs at the activation loop, turn motif, and hydrophobic motif. These latter sites are involved in the maturation of PKC rendering it capable of being activated upon ligand binding. We therefore only examined activation of PKCδ in response to I3A and PMA. Other PKC isoforms reported for Ramos cells include PKCα, β, ε, and θ [32].

**Figure 2:**
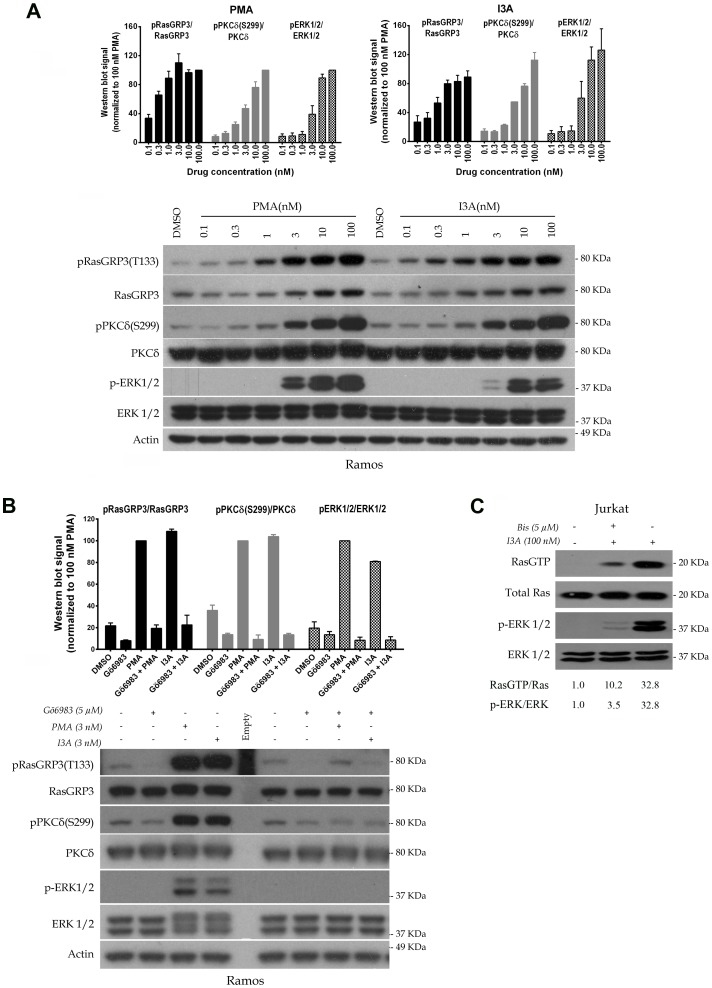
Comparison of the potencies of I3A and PMA for biochemical responses at the level of induced protein phosphorylation. A. Ramos cells were treated for 30 minutes with the indicated concentrations of I3A or PMA followed by analysis of cell lysates by immunoblotting. DMSO indicates the vehicle control. Bars indicate quantitation (mean ± SEM) of the results from three independent experiments. A representative immunoblot is illustrated. It should be noted that although the RasGRP3 antibody is not directed against a RasGRP3 phosphorylation site, it consistently yields some increase in signal under conditions of RasGRP3 phosphorylation. B. Ramos cells were treated with 3 nM I3A or PMA, in some cases after pretreatment with Gö6983 (5 µM for 40 min), and analyzed as above. Bars indicate quantitation (mean ± SEM) of the results from two independent experiments. A representative immunoblot is illustrated. An additional experiment performed under similar conditions gave similar results. C. Jurkat T cells were stimulated in a single experiment with I3A for 10 min with or without 10 min pre-incubation with the pan-PKC inhibitor bisindolymaleimide I (4.6 µM), followed by analysis of Ras-GTP, total Ras, pErk1/2 and total Erk1/2 levels.

In this cell system, we observed that I3A and PMA showed approximately equal potencies for activation of PKC δ and for Erk (Fig. 2A). RasGRP3 phosphorylation at T133 was detectable at a slightly lower concentration of either ligand, suggesting the involvement of other PKC isoforms in addition to PKCδ. To confirm the involvement of PKC in RasGRP3 phosphorylation, we examined the ability of the general PKC inhibitor Gö6983 to block these phosphorylation events (Fig. 2B). By itself, pretreatment with Gö6983 had little effect. In contrast, the responses to treatment with I3A or PMA were dramatically decreased. In particular, phosphorylation of RasGRP3 at T133 was almost fully abolished, consistent with the inhibition of PKCδ phosphorylation, as was the downstream response of Erk phosphorylation. Likewise, in Jurkat T cells the pan PKC inhibitor bis-indolemaleimide I abolished the downstream response to I3A of Erk phosphorylation (Fig. 2C). Furthermore, we confirmed that this PKC inhibitor greatly diminished the formation of Ras-GTP in response to I3A, directly demonstrating that the effect of I3A on Erk phosphorylation was upstream of Ras and consistent with the known requirement for PKC-mediated activation of RasGRP1 in addition to the requirement for ligand binding to the RasGRP1 C1 domain [15], [18], [19].

### I3A Activates RasGRPs in Select B-NHL Cell Lines

**Figure 3:**
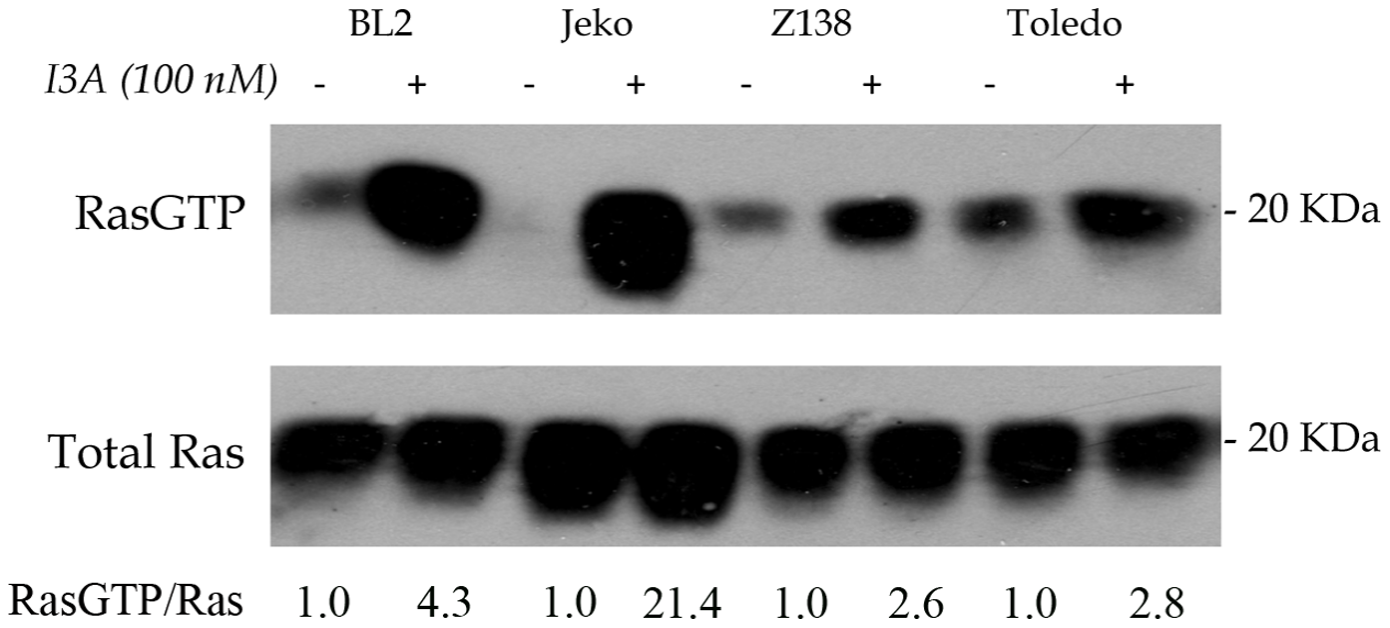
Activation of Ras by I3A in representative B-NHL cell lines BL2, Jeko-1, Z138 and Toledo. In a single experiment, cells were treated with 100 nM I3A or DMSO control for 10 minutes, followed by analysis of Ras-GTP by the pull down assay. The ratio of Ras-GTP/Ras was quantitated.

We previously showed that DAG analogues such as bryostatin 1 and the synthetic analogue pico could activate RasGRPs in B-NHL cell lines that have been characterized as sensitive to induction of apoptosis by these same compounds [25], [26]. Having confirmed above that I3A acted as a DAG analogue on RasGRP1/3, we wished to explore in more detail the ability of this compound to elicit Ras activation in representative DAG analogue sensitive B-NHL cell lines. Brief treatment of BL2 (Burkitt’s lymphoma), Jeko-1, Z138 (both mantle cell lymphoma) and Toledo (germinal center lymphoma) resulted in robust activation of Ras (Fig. 3).

### I3A Treatment Causes Intracellular Re-distribution of RasGRP1 and RasGRP3

DAG analogues bind to a hydrophilic cleft in the C1 domain, completing a hydrophobic surface and thereby driving membrane insertion of the DAG analogue – C1 domain complex [33]. DAG and DAG analogues thus contribute to RasGRP activation in part by causing these proteins to re-locate to cellular membranes where they can physically interact with lipidated Ras [34]. We therefore examined the ability of I3A to induce protein redistribution using both *in situ* and subcellular fractionation approaches.

**Figure 4:**
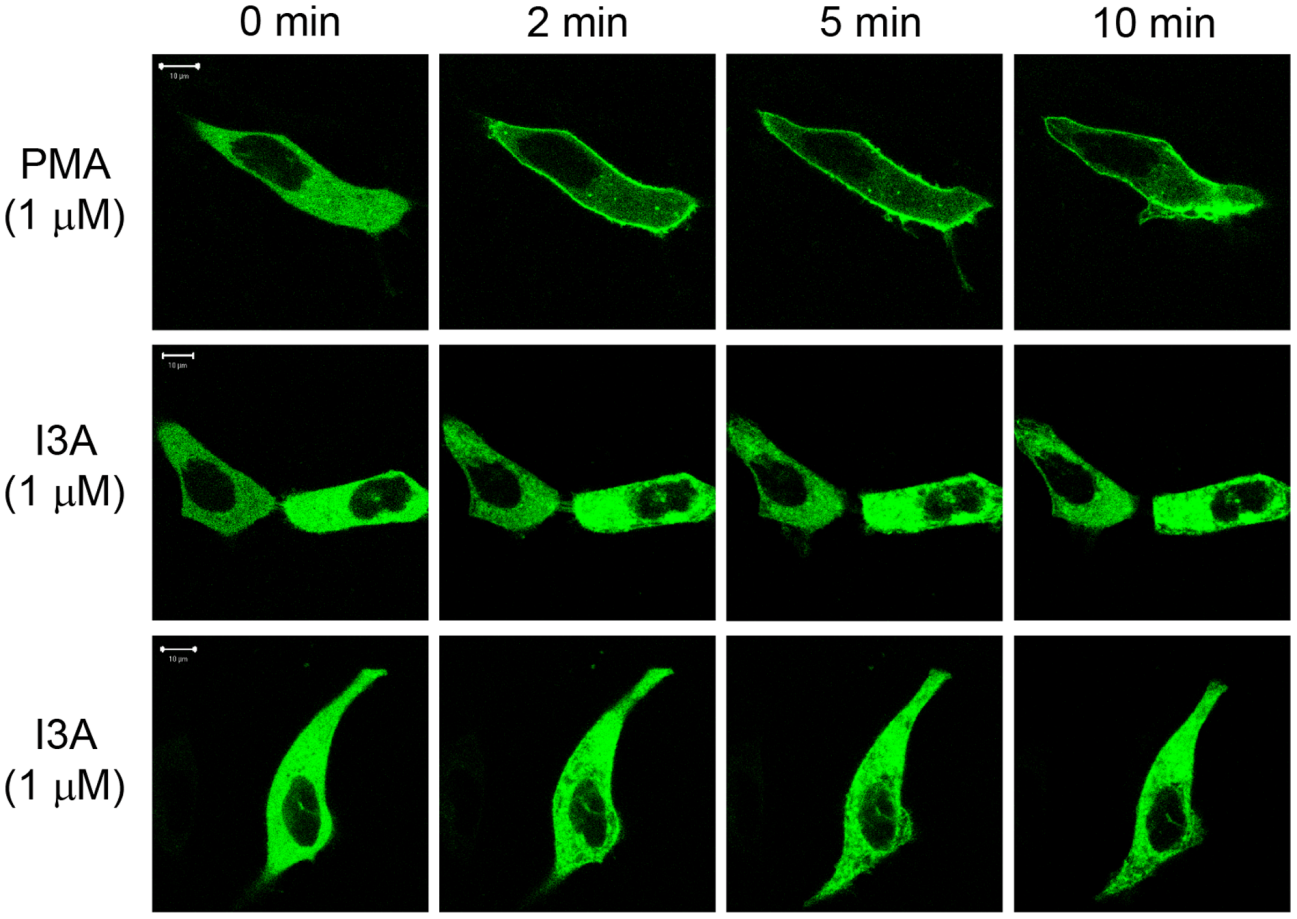
Translocation of GFP-tagged RasGRP1 in living cells after treatment with PMA or I3A. Cells expressing GFP-RasGRP1 were treated with 1000 nM PMA or I3A. The translocation pattern was examined in live cells as a function of time. The results of duplicate experiments with I3A are shown. The images shown are representative of four independent experiments. The scale bar represents 10 µm.

For *in situ* studies, GFP-RasGRP1 was expressed in LNCaP cells. After treatment with I3A or PMA, changes in the intracellular pattern of labeled protein were visualized by confocal microscopy. LNCaP cells were chosen because their well-spread morphology facilitates imaging. They are easy to transfect and we have had extensive experience with the effects of various phorbol esters on PKC translocation in this system [29]. Before treatment, GFP-RasGRP1 was distributed throughout the cytoplasm (Fig. 4). Upon PMA (1000 nM) addition, GFP-RasGRP1 translocated to the plasma membrane within 2 minutes. I3A likewise induced a rapid response, but the pattern was markedly different, with pronounced clustering at internal sites.

**Figure 5:**
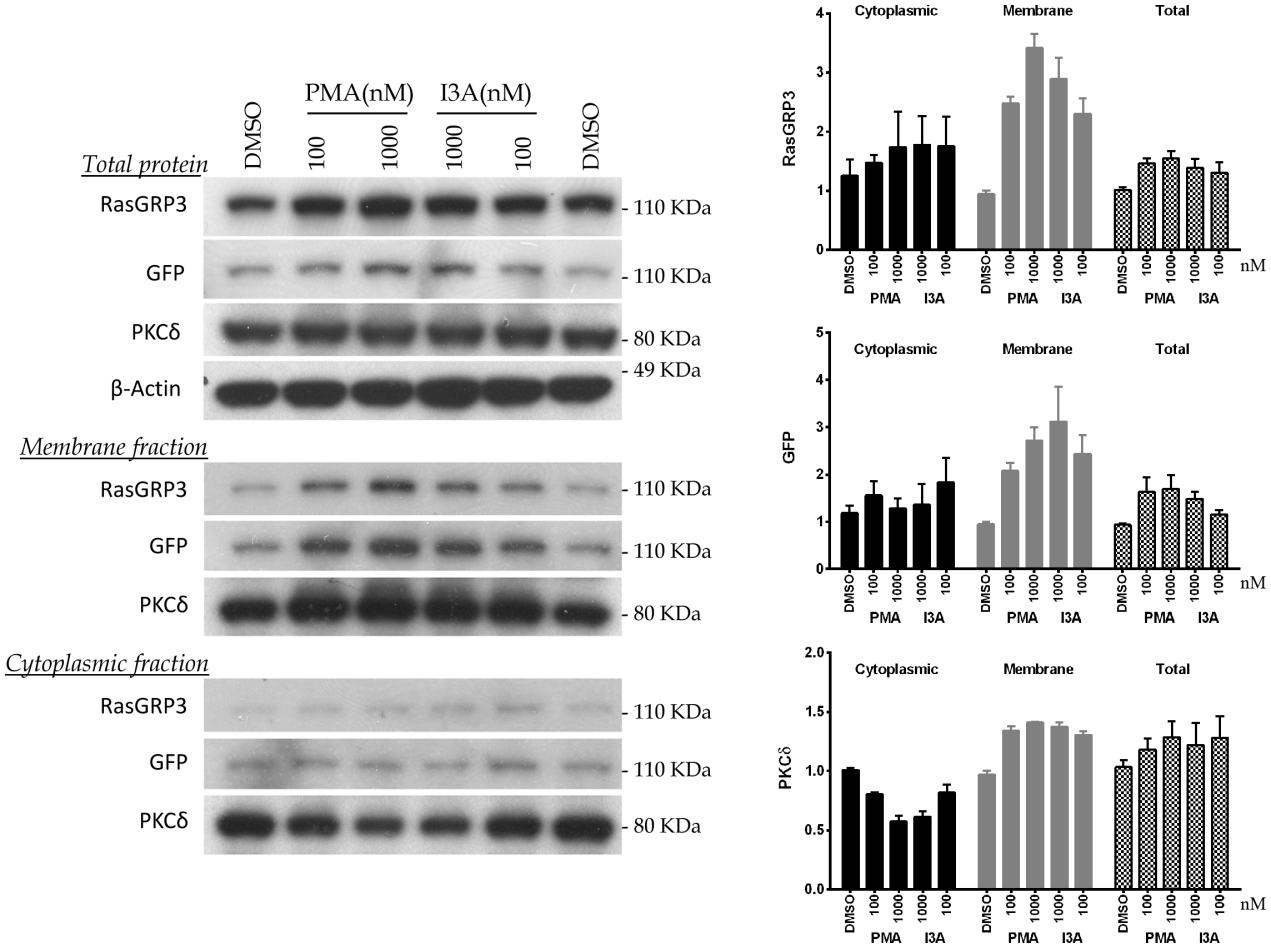
Changes in subcellular distribution of RasGRP3 and PKCδ in response to treatment with PMA or I3A. HEK-293 cells expressing GFP-RasGRP3 were treated for 30 minutes with PMA or I3A at the indicated concentrations. Cell sonicates were subjected to subcellular fractionation and the levels of PKCδ and RasGRP3 were analyzed by immunoblotting. For RasGRP3, expression was detected both with an anti-RasGRP3 antibody and with an antibody against the GFP tag. Bars indicate quantitation (mean ± SEM) of the results from three independent experiments. A representative immunoblot is illustrated.

As a complementary approach, we expressed GFP-RasGRP3 in HEK-293 cells, treated with I3A or PMA, subjected the cells to subcellular fractionation, and examined the shifts in distribution of GFP-RasGRP3 between cytoplasmic and membrane fractions (Fig. 5). GFP-RasGRP3 was detected both with an antibody directed against RasGRP3 itself as well as with an antibody against the GFP tag. For comparison, we also examined shifts in distribution of endogenous PKCδ.

Treatment with I3A induced a clear increase in the level of GFP-RasGRP3 recovered in the membrane fraction, detected with either antibody, resembling the response observed with PMA. The loss of GFP-RasGRP3 from the cytoplasmic fraction was less obvious, but these results should be tempered by the observation that protein detected in the total protein sample was slightly elevated in DAG analogue treated samples, for unknown reasons. In the same experiment, both I3A and PMA induced redistribution of PKCδ to the membrane fraction.

### I3A Treatment Affects Bcl-2 Proteins in Select B-NHL Cell Lines

**Figure 6:**
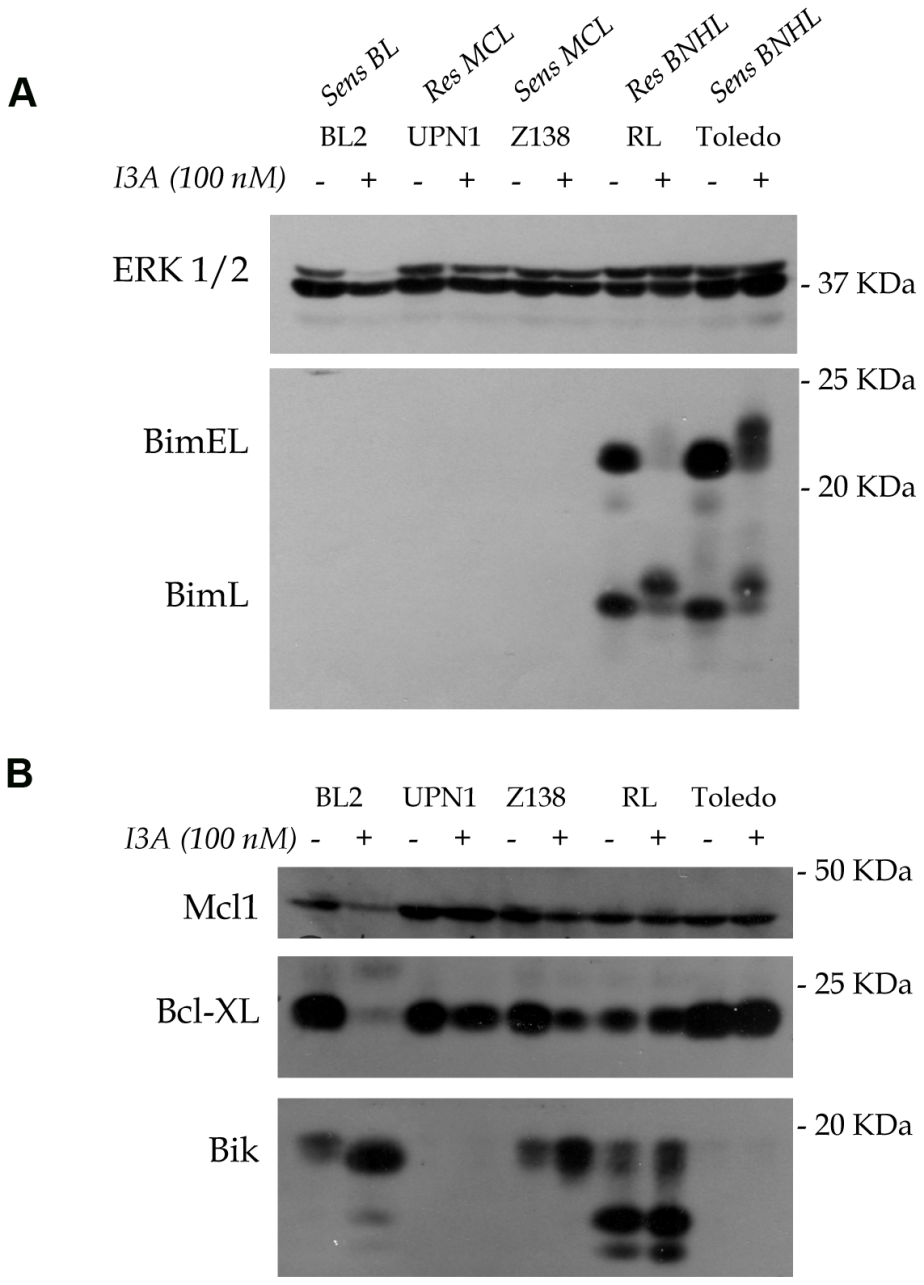
Quantitative and qualitative changes in Bcl-2 family members induced by I3A in representative B-NHL cells. The indicated cell lines were untreated or treated for 3 days (BL2, UPN1, Z138) or for 24 hours (RL and Toledo). Protein lysates were analyzed by immunoblotting with antibodies to the indicated proteins. Panels A and B were derived from duplicate gels. Erk was used as a loading control.

In our previous studies we compared the DAG analogue-sensitive cell line Toledo to RL, a DAG analogue-resistant germinal center type B-NHL cell line [25]. We reported that apoptosis induced by DAG analogues in Toledo B-NHL cells was dependent on the pro-apoptotic BH3-only protein Bim [25]. In Toledo B-NHL cells, treatment with bryostatin 1 or pico resulted in RasGRP-Erk signaling and phosphorylation of Bim on a pro-apoptotic site that is common to both BimEL and BimL splice forms. We also showed that anti-apoptotic phosphorylation on sites unique to BimEL led to efficient proteolysis in a proteosome-dependent manner in the DAG analogue-resistant cell line RL. However, this anti-apoptotic mechanism was less evident in Toledo. Our present results with I3A show exactly the same pattern: BimL assumed a reduced electrophoretic mobility, indicative of phosphorylation, after I3A treatment in both RL and Toledo (Fig. 6A). I3A also elicited phosphorylation of BimEL in both RL and Toledo. However, BimEL down-regulation in Toledo was relatively inefficient after I3A treatment (Fig. 6A), just as we discovered previously for bryostatin 1 and pico [25].

More recently we showed that pico treatment induced apoptosis in the Burkitt’s lymphoma cell line BL2 and in 5 of 6 mantle cell lymphoma cell lines, most of which were Bim-deficient [26]. In these cases, apoptosis was associated with increased expression of Bik and decreased expression of Mcl1 and Bcl-XL. In the present studies, we found that Bik was induced by I3A treatment in BL2 and Z138, a representative mantle cell lymphoma cell line (Fig. 6B). Especially in BL2, much of the expressed Bik had increased electrophoretic mobility. This phenomenon, which may reflect alternate mRNA splicing or post-translational modification, was previously seen in pico-treated cells [26]. In contrast, the DAG analogue-resistant UPN1 MCL cell line did not exhibit I3A-induced Bik expression. Both BL2 and Z138 showed IA3-induced decreased expression of Mcl1 and Bcl-XL, but these effects were not as evident in UPN1 cells (Fig. 6B). In every respect, the results obtained here with I3A parallel those obtained with bryostatin 1 and its analogue pico in our previous studies [25], [26].

### I3A Induces Apoptosis in Select B-NHL Cell Lines

**Figure 7:**
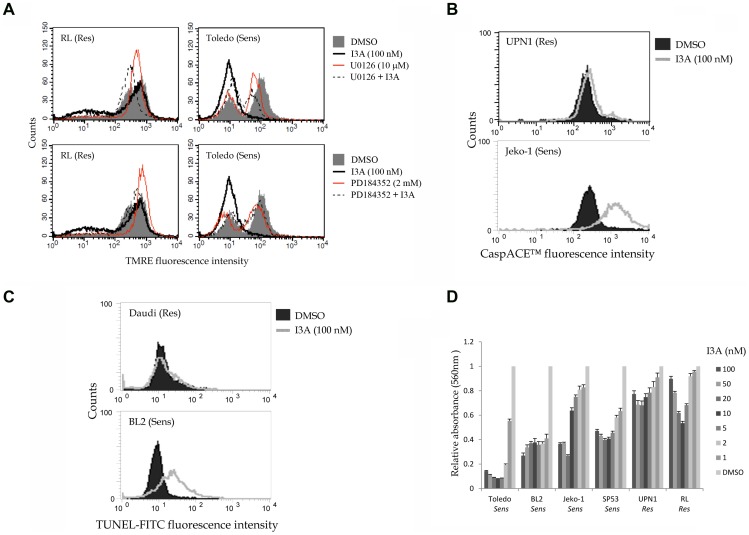
Representative experiments showing induction of apoptosis and inhibition of viable cell proliferation by I3A in B-NHL cells. A. RL and Toledo cells were treated for 24 hrs, incubated with TMRE, and analyzed for stain uptake by flow cytometry. In some cases the cultures were incubated with either U0126 or PD184352 to assess the effects of Mek inhibition. B. UPN1 and Jeko-1 cells were incubated with DMSO or 100 nM I3A for 4 days and then analyzed with CaspACE™ to detect caspase activation. C. Daudi and BL2 cells were incubated with DMSO or I3A for 4 days and then assayed for nuclear DNA fragmentation using TUNEL. For A-C, representative data are shown. D. Cells were cultured in 96 well plates for 2 days (RL and Toledo) or 7 days (other cell lines) in regular medium or medium containing the indicated concentrations of I3A. Viable cell accumulation was quantified using the MTT assay. The absorbance value of DMSO treated control cells was set to 1.0 for comparison. The results for all cell types are summarized in Table 2.

To determine whether I3A can induce apoptosis in B-NHL cell lines previously shown to be sensitive to bryostatin 1 and/or its analogue pico, we used three different cell labeling protocols and flow cytometry, as previously described [25], [26]. Besides the cell lines mentioned above, we included in the analysis the previously characterized DAG-analogue-sensitive MCL cell lines Mino, Rec1, and Sp53 and the resistant Burkitt’s lymphoma cell line Daudi. The inability of cells to accumulate TMRE (tetramethylrhodamine ethyl ester) was used to detect loss of the mitochondrial outer membrane potential (Fig. 7A). In some cases, we included the Mek inhibitors U1026 or PD184352 to assess the role of the canonical Ras signaling pathway in apoptosis. A fluorescent caspase pseudo-substrate was used to detect global caspase activity (Fig. 7B). DNA end-labeling using dUTP and terminal transferase (TUNEL) was employed to monitor nuclear DNA fragmentation (Fig. 7C). Representative findings are presented in Figure 7 and the results are summarized in Table 2. In general, I3A induced extensive apoptosis in DAG analogue-sensitive cells lines (Toledo, BL2, Jeko-1), but much less so in resistant cells lines (RL, UPN1 and Daudi). Loss of TMRE staining induced by I3A in Toledo cells was largely abrogated by Mek inhibitors (Fig. 7A), but this effect was less dramatic in some of the MCL cell lines (data not shown).

**Table 2:** Effects of I3A on apoptosis and viable cell accumulation in B-NHL cell lines.

	TMRE(#)			CaspACE™(#)			TUNEL(&)			MTT(∧)
*Cell Line*	*DMSO*	*I3A*	*fold*	*DMSO*	*I3A*	*fold*	*DMSO*	*I3A*	*fold*	*IC_50_*
		*(100 nM)*	*increase*		*(100 nM)*	*increase*		*(100 nM)*	*increase*	
**BL2(BL)**	12.02**	95.01**	7.90	8.03*	12.62*	1.57	5.945*	92.48*	*15.56*	5.49
**Daudi (BL)**	12.14**	21.62**	1.78	19.79	48.17	2.43	3.52	1.16	0.33	>100
**Jeko (MCL)**	4.18**	42.47**	10.16	1.91	68.15	35.68	1.86	61.23	32.92	3.22
**Mino (MCL)**	10.16*	95.73*	9.42	0.75	9.29	12.39	0.29*	84.39*	291.00	4.07
**Rec1 (MCL, $)**	–	–	–	2.58	24.51	9.50	7.76*	57.23*	7.38	3.18
**SP53 (MCL)**	14.085*	90.14*	6.40	1.17	3.69	3.15	0.74	91.85	124.12	3.75
**UPN1 (MCL)**	14.01**	23.59**	1.68	0.85	0.99	1.16	1.55*	3.00*	1.94	>100
**Z138 (MCL)**	7.52**	46.38**	6.17	3.55*	10.48*	2.95	5.63	17.90	3.18	3.34
**RL (GC)**	26.39**	41.47**	1.57	3.81*	14.46*	3.80	0.59	9.16	15.53	>100
**Toledo (GC)**	14.31**	96.24**	6.73	1.55*	9.46*	6.10	8.35	79.67	9.54	6.65

TMRE, the percent of cells incapable of assimilating TMRE is shown.

CaspACE, the percent of cells showing activated caspases is shown.

TUNEL, the percent of cells showing nuclear DNA fragmentation is shown.

MTT, the concentration of I3A that caused 50% inhibition of viable cell accumulation was calculated by linear regression.

# for TMRE and CaspACE assays cells were treated with 100 nM I3A for 4 days, except RL and Toledo were treated for 24 hr.

&, for TUNEL assay cells were treated with 100 nM I3A for 7 days, except RL and Toledo were treated for 2 days.

* average of two experiments.

** average of three experiments.

$ Rec1 cells do not stain with TMRE [25].

Finally, we used the MTT assay to determine the relationship between I3A dose and accumulation of viable cells using representative drug-resistant and drug-sensitive cell lines (Fig. 7D). Cell lines previously shown to be sensitive to bryostatin 1 and/or pico were unable to propagate even in very low nano-molar concentrations of I3A. In contrast, accumulation of viable drug-resistant cell lines such as UPN1, RL and Daudi was less impaired by I3A (Fig. 7D, Table 2). Interestingly, evidence for a bimodal dose-response was evident in some cell lines.

## Discussion

I3A binds the C1 domain of PKCs and is a known activator of PKCs. In this study we show that I3A also binds and activates RasGRP1 and RasGRP3. Either of these RasGRPs expressed ectopically in Rat2 cells endows the cells with the ability to respond to I3A in a fashion similar to that of PMA. In addition, I3A activates Ras in Jurkat T cells, a cell line known to depend on RasGRP1 for coupling of DAG signaling to Ras. Thymocytes depend on RasGRP1 to activate Ras in response to PMA or bryostatin 1 [27]. Here we show that robust Erk phosphorylation in response to either PMA or I3A is absent in thymocytes deficient in RasGRP1. Importantly, I3A robustly activates Ras in B-NHL cell lines previously shown to express RasGRPs and to exhibit DAG analogue-induced, RasGRP-stimulated apoptosis.

In Jurkat T cells, I3A-induced Ras and Erk activation was muted in the presence of a PKC inhibitor, bis-indolemaleimide I. Likewise, we showed that RasGRP3 in Ramos B cells undergoes regulatory phosphorylation on T133 in response to either I3A or PMA. The PKC inhibitor Gö6983 greatly diminished both RasGRP3 phosphorylation and downstream signaling to Erk. In these studies we examined the behavior of PKCδ in parallel and obtained evidence that this protein, which may contribute to RasGRP3 phosphorylation, behaved similarly to RasGRP3 in terms of its response to either PMA or I3A and the PKC inhibitor Gö6983. A complication in these studies arises from the fact that the intensity of our immunoblot signals obtained with the anti-RasGRP3 antibody reproducibly was higher when proteins were derived from DAG analogue treated cells, despite the fact that the antibody was not directed towards a phospho-epitope. This effect has been observed with two independent anti-RasGRP3 antibodies and seems unlikely to be accounted for by increased protein expression as the treatment times are very short. Collectively, our results show that I3A activates RasGRP1 and RasGRP3 signaling and support the idea that PKC-mediated phosphorylation may be involved, as previously demonstrated for PMA. The known dependence of RasGRP1/3 on its C1 domain for response to DAG analogues [15], however, makes clear that the functional response of RasGRP1/3 reflects both the direct interaction of I3A with the RasGRP1/3 as well as the indirect effect through PKC activation and RasGRP1/3 phosphorylation.

*In situ* studies with GFP-tagged RasGRP1 show that I3A can drive the membrane translocation of RasGRP1. Notably, I3A was less effective than PMA at directing RasGRP1 to the plasma membrane. Previously we showed that PMA, I3A and other C1 domain ligands exhibited different abilities to translocate various PKCs including PKCδ to different membranes [29], [9], [35]. Importantly, like I3A, the non-tumor promoting DAG analogue bryostatin 1 was shown to preferentially target RasGRP1 to internal membranes, compared to PMA [36]. We also showed here that I3A, like PMA, can effect translocation of full-length RasGRP3 to the membrane fraction using subcellular fractionation methods. These results thus provide further confirmation that I3A activates RasGRPs through membrane recruitment, through direct binding of membrane-associated I3A to their C1 domains, in concert with PKC-mediated phosphorylation. Understanding the membrane specificity of different C1 domain proteins and their ligands may be key to dissociating tumor-promoting versus anticancer effects of DAG analogues [35].

We previously showed that DAG analogues such as bryostatin 1 and pico could induce apoptosis in B-NHL cell lines by at least two distinct mechanisms. In Toledo cells, apoptosis is relatively rapid, depends on Bim and is associated with phosphorylation of Bim on pro-apoptotic sites, as well as relatively inefficient BimEL proteolysis [25]. In contrast, apoptosis in the Burkitt’s-derived cell line BL2 and 5 of 6 MCL cell lines, typified by Z138, proceeds much more slowly. This latter mechanism of apoptosis occurs in the absence of Bim and is associated with quantitative changes in Bik, Mcl-1 and Bcl-XL that favor cell death, as well as a qualitative change in Bik of unknown significance. In the present study, we show that I3A induces all the Bcl-2 family member changes previously documented. It may also be noteworthy that the effects on Bik, Bcl-XL and Mcl1 documented recently in BL2 and MCL cell lines, and confirmed here, were not observed in Toledo cells, supporting our previous proposal that the documented protein changes are specific to the two kinetically distinct apoptotic mechanisms [26].

We also showed here that I3A potently induces apoptosis in sensitive cell B-NHL lines, but substantially less apoptosis in cell lines previously characterized as DAG analogue-resistant. Loss of TMRE staining, which is an early marker of apoptosis by a mitochondrial pathway, was diminished in the presence of Mek inhibitors in some cases, implicating the RasGRP-Erk signaling axis in apoptotic signaling (Fig. 7A).

RasGRPs are expressed in T and B lymphocytes, mast cells, neurons, myeloid precursors, skin keratinocytes and endothelial cells [12]. Some of the anti-cancer actions documented with I3A might arise from activation of RasGRPs. In particular I3A-induced apoptosis in AML cells was associated with prolonged Erk activation [37] and might depend on RasGRP1 or RasGRP4 signaling. I3A induces senescence in a fraction of melanoma cell lines by a Mek-dependent mechanism [38], [5]. At least one of the melanoma cell lines in this latter study and a fraction of primary melanomas were recently shown to express RasGRP3 [39]. I3A activation of tumoricidal cytotoxic T lymphocytes [8] likely involves RasGRP1. I3A also promotes recruitment of neutrophils to tumors and this process is thought to involve activation of vasculature endothelial cells [7], [40]. Intriguingly, topically applied I3A was reported to disrupt the vasculature system [6], a property reminiscent of the ability of PMA to disrupt angiogenesis in a RasGRP3-dependent manner [41]. Besides sharing properties with other DAG analogues, I3A has unique characteristics that might be important for maximizing its potential as an anti-cancer compound. For example, compared to PMA, I3A shows remarkable skin penetration properties, which may reflect its ability to translocate between cells by way of the epidermal multidrug transporter [6]. I3A is also remarkable in that it is present at a clinically relevant concentration in the sap of a commonly available shrub. As recognized by early herbalists, the sap of *Euphorbia peplus* is effective at treating non-melanoma skin cancer [42].

The present studies lay the groundwork for understanding I3A action in terms of well-researched Ras signaling systems. They additionally lend credence to our previous proposal that RasGRPs constitute viable drug targets. I3A joins a growing list of DAG analogues that might be used to target RasGRPs in a clinical setting.
